# Recent Trends in Machine Learning, Deep Learning, Ensemble Learning, and Explainable Artificial Intelligence Techniques for Evaluating Crop Yields Under Abnormal Climate Conditions

**DOI:** 10.3390/plants14182841

**Published:** 2025-09-11

**Authors:** Ji Won Choi, Mohamad Soleh Hidayat, Soo Been Cho, Woon-Ha Hwang, Hoonsoo Lee, Byoung-Kwan Cho, Moon S. Kim, Insuck Baek, Geonwoo Kim

**Affiliations:** 1 Department of Biosystems Engineering, College of Agricultural and Life Sciences, Gyeongsang National University, 501, Jinju-daero, Jinju 52858, Republic of Korea; jiwonbb626@gnu.ac.kr (J.W.C.); hdytsoleh@gmail.com (M.S.H.); bpagoe@gmail.com (S.B.C.); 2 Division of Crop Production and Physiology, National Institute of Crop Science, Rural Development Administration, 100, Nongsaengmyeong-ro, Iseo-myeon, Wanju-gun 55365, Republic of Korea; hwangwh@korea.kr; 3 Department of Biosystems Engineering, College of Agriculture, Life, and Environment Sciences, Chungbuk National University, 1 Chungdae-ro, Seowon-gu, Cheongju 28644, Republic of Korea; 4 Department of Smart Agricultural System, Chungnam National University, Daehak-ro, Daejeon 34134, Republic of Korea; chobk@cnu.ac.kr; 5 Department of Biosystem Machinery Engineering, Chungnam National University, Daehak-ro, Daejeon 34134, Republic of Korea; 6 Environmental Microbial and Food Safety Laboratory, Agricultural Research Service, United States Department of Agriculture, Powder Mill Road, BARC-East, Bldg 303, Beltsville, MD 20705, USA; moon.kim@usda.gov (M.S.K.); insuck.baek@usda.gov (I.B.); 7 Institute of Smart Space Agriculture, Gyeongsang National University, 501, Jinju-daero, Jinju 52828, Republic of Korea

**Keywords:** crop yield, machine learning, deep learning, artificial intelligence, abnormal climate

## Abstract

Crop yield prediction (CYP) has become increasingly critical in addressing the adverse effects of abnormal climate and enhancing agricultural productivity. This review investigates the application of advanced Artificial Intelligence (AI) techniques including Machine Learning (ML), Deep Learning (DL), Ensemble Learning, and Explainable AI (XAI) to CYP. It also explores the use of remote sensing and imaging technologies, identifies key environmental factors, and analyzes the primary causes of yield reduction. A wide diversity of input features was observed across studies, largely influenced by data availability and specific research goals. Stepwise feature selection was found to be more effective than increasing feature volume in improving model accuracy. Frequently used algorithms include Random Forest (RF) and Support Vector Machines (SVM) for ML, Artificial Neural Networks (ANNs) and Convolutional Neural Networks (CNNs) for DL, as well as stacking-based ensemble methods. Although XAI remains in the early stages of adoption, it shows strong potential for interpreting complex, multi-dimensional CYP models. Hyperspectral imaging (HSI) and multispectral imaging (MSI), often collected via drones, were the most commonly used sensing techniques. Major factors contributing to yield reduction included atmospheric and soil-related conditions under abnormal climate, as well as pest outbreaks, declining soil fertility, and economic constraints. Providing a comprehensive overview of AI-driven CYP frameworks, this review offers insights that support the advancement of precision agriculture and the development of data-informed agricultural policies.

## 1. Introduction

According to the FAO Report (FAO, 2023, https://www.fao.org/newsroom/detail/first-ever-global-estimation-of-the-impact-of-disasters-on-agriculture/en (accessed on 7 August 2024)), the average loss of agricultural products due to disasters related to abnormal climate amounts to $123 billion per year, or five percent of the annual global agricultural gross domestic product (GDP). Abnormal climate refers broadly to weather and climate phenomena that deviate from the normal range of climatic variability and negatively affect agricultural productivity [1]. Specifically, it encompasses various climatic factors such as droughts, heatwaves, floods, irregular precipitation, and cold damage, which disrupt crop growth conditions and lead to reductions in both yield and quality [2].

Environmental conditions greatly affect yield production of field crops. The main causes of declines in crop production yields are abnormal climate conditions that cause agricultural land to experience extreme low rainfall and high temperatures [3]. Abnormal climate not only reduces crop yield production, but also leads to declines in the quality of agricultural products [4,5]. To address this problem, technology than can evaluate and predict crop yield while maintaining sustainable farming practices is needed [6].

Crop yield evaluation is an important task in the modern agricultural era, considering today’s unstable environmental conditions [7]. In the past, crop yield prediction (CYP) was primarily conducted using linear regression or statistical models based on historical numerical data such as climate conditions, soil properties, and cultivated area [8,9]. Although these methods were simple and easy to interpret, they had limitations in adequately capturing the complex interactions among various factors such as abnormal climate, pest outbreaks, and agricultural management practices [10]. In addition, incorporating real-time data was difficult and the methods were limited in responding to exceptional situations, and in this light they were insufficient for precise prediction and timely decision-making [11]. However, since the 2000s, the introduction of Machine Learning (ML) and Deep Learning (DL) models has significantly improved prediction accuracy, with the root-mean-square error (RMSE) reduced to approximately 1.2 tons per hectare [12].

More recently, smart agriculture platforms that integrate real-time monitoring of soil moisture and weather conditions through Internet of Things (IoT) sensors, along with UAV imagery, have emerged, enabling AI to support immediate decision-making for irrigation and fertilization [13,14]. At the same time, block chain-based data governance frameworks are being implemented to prevent data tampering and enhance traceability throughout the supply chain [15].

ML is a branch of computer science that is based on the theoretical study of pattern recognition and automatic learning related to artificial intelligence (AI), and it is widely used to predict and analyze data [16]. In the past, the field of ML focused primarily on algorithms and optimization theory, but today it encompasses a wide range of disciplines such as statistics, information theory, probability, and functional analysis [17]. ML technologies are also being used to create predictive models, methods for real-world applications, and optimization techniques [18].

ML techniques have recently been used to predict crop yields under abnormal climate [19]. Crop yield is a nonlinear and time-dependent issue influenced by a complex interplay of spatial, climatic, and environmental factors, and accordingly it is difficult for traditional, simple statistical approaches to effectively capture this complexity [20]. ML is recognized as a powerful technique capable of improving prediction accuracy by learning the complex interactions among various environmental factors, and it can maintain relatively high performance even under highly uncertain conditions such as abnormal climate [21,22].

DL models, alongside ML techniques, are increasingly attracting attention in the field of CYP [23]. DL effectively handles complex data through hierarchical representation learning enabled by deep network architectures and demonstrates strong performance in analyzing and predicting various types of unstructured data arising from abnormal climate [24]. In addition, DL can be applied in both online and offline environments and can be effectively integrated with agricultural automation and real-time response systems [25]. However, its implementation requires certain conditions, as it depends on large-scale datasets and high-performance computational resources [7,26].

Ultimately, to effectively address crop yield reductions caused by abnormal climate, intelligent prediction systems based on ML and DL should be adopted, while also enhancing model generalizability and prediction reliability through training and application across diverse environmental conditions [27,28]. In addition, ensemble learning and explainable artificial intelligence (XAI) are widely utilized to enhance both the performance and interpretability of AI models [29].

Ensemble learning combines the predictions of multiple models instead of relying on a single model. This improves overall accuracy by compensating for the weaknesses of individual models [30]. Common techniques include bagging, boosting, and stacking, which reduce data variance, correct model errors, and integrate diverse models, respectively [31]. These methods are particularly effective in complex and high-dimensional environments, where they help mitigate overfitting and enhance prediction consistency [32]. Accordingly, such techniques have recently been employed in crop yield prediction research [33,34].

XAI is a technology designed to clarify the decision-making process of AI models and resolve the so-called “black box” problem [35]. This is especially critical in practical applications such as agriculture, where users should understand and trust AI predictions in order to incorporate them into decision-making [36]. XAI enhances transparency by either improving the interpretability of model structures or employing post hoc explanation methods, such as LIME and SHAP, which visually illustrate the contribution of each input feature to the prediction [37]. These techniques strengthen the interaction between the user and the model. As a result, XAI techniques have been applied to crop yield prediction in recent studies in order to better understand the factors driving AI model decisions [38,39,40].

In conclusion, while ensemble learning focuses on improving prediction accuracy and robustness, XAI enhances the reliability and transparency of AI systems. Together, they serve as key components in making AI-based crop yield prediction systems both practical and trustworthy for real-world implementation.

Crop yield is a complex outcome influenced by the combined effects of various environmental factors such as climate, soil conditions, moisture levels, temperature, and pest infestations [41]. Due to recent abnormal climatic conditions, even the same crop may exhibit different growth responses depending on the region and timing; AI-based prediction models therefore must be trained to reflect a wide range of environmental conditions [42]. By accounting for such environmental diversity, the model’s generalization performance can be improved, while also enhancing the reliability and interpretability of its predictions [43]. Therefore, to enhance the accuracy and practical applicability of AI-based CYP, it is essential to investigate environmental factors systematically and to collect integrated, comprehensive datasets [44]. Furthermore, sensor technologies and remote sensing data that quantitatively capture environmental information can play a crucial role in enhancing the precision of AI models and enabling real-time responsiveness [45,46].

To ensure consistent performance across diverse environments, AI models must be trained to incorporate various environmental conditions. Incorporating environmental factors into model training greatly enhances the generalization ability of the model, thereby contributing to improved performance and efficiency of ML and DL. The unique contribution of this study lies in its focus on crop yield prediction under abnormal climate conditions, which has been insufficiently addressed in previous research. To support this, the study includes a comprehensive review of the latest trends such as ensemble learning and XAI, as well as a quantitative meta-analysis based on performance metrics (e.g., RMSE, R^2^). Therefore, the objectives of this study are to summarize the specific characteristics of ML, DL, ensemble learning, and XAI techniques, along with imaging-based data acquisition methods, analyze the impacts of abnormal climate and environmental factors on crop yield prediction, and systematically synthesize and critique existing studies to propose future research directions. To achieve these objectives, a systematic literature review (SLR) was conducted on studies that applied AI and imaging techniques to CYP.

## 2. Article Search Strategy

Using Google Scholar, Scopus, Web of Science, and Science Direct, we selectively searched for the following keywords that are closely related to this study: Machine learning, Deep learning, Yield prediction, Crop, Artificial Intelligence, Abnormal climate, etc., in published journal and conference papers (2019~2024) in the last five years. After filtering, approximately 100 papers were collected and categorized as given below. Among these, only studies explicitly or implicitly related to abnormal climate conditions were selected for further analysis.

- Types and examples of ML used to predict crop yield;
- Types and examples of DL utilized to predict crop yields;
- Ensemble learning types and examples used to predict crop yields;
- Types and examples of XAIs used to predict crop yields;
- Imaging devices used for crop yield prediction;
- Environmental factors affecting crop yields;
- Other causes of crop yield reduction.

We also set the boundaries of the systematic review, using exclusion criteria (EC) for irrelevant studies. The EC were as follows:

- EC 1—the publication is not written in English;
- EC 2—the publication is a duplicate or already searched for;
- EC 3—the full text of the publication is not available;
- EC 4—the publication is a survey;
- EC 5—if the publication was published before 2018.

**Figure 1:**
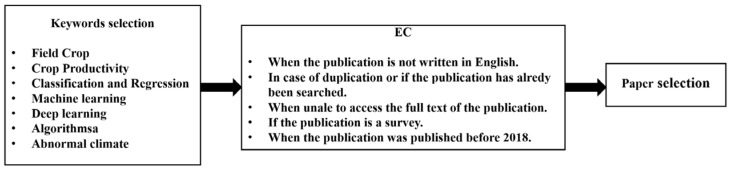
Paper selection criteria.

Finally, approximately sixty papers were selected after applying the EC, and the selected papers were mainly published by Elsevier, Springer, MDPI, etc. (Figure 1, Table 1).

## 3. AI Model Development for CYP

AI-driven CYP models are typically composed of three main stages: data collection, data preprocessing, and AI-based prediction. In the data collection stage, various sensing technologies such as hyperspectral imaging (HSI), multispectral imaging (MSI), RGB imaging, and thermal imaging (TRI) are utilized to acquire diverse information related to crop growth conditions, disease incidence, moisture levels, and environmental factors [46,47]. Among them, HSI and MSI provide high-resolution spectral data that enable precise analysis of physiological traits, whereas RGB imaging is not able to obtain spectral data [47,48]. However, it provides cost-effective, rapid scanning speed over large areas [49]. TRI is employed to capture temperature variations across crop fields, which are critical for assessing plant stress and evapotranspiration [50].

After the data acquisition stage, the preprocessing stage is essential because environmental noise and data distortion should be removed from the obtained data and spectral/spatial calibration should be performed to enhance the raw data quality [51]. The preprocessing stage is composed of a series of refinement steps, including the handling of missing values, outlier removal, normalization, image augmentation, computation of vegetation indices, and so on [52]. Furthermore, spatial alignment and seamless integration of multi-sensor data can be achieved for effective model training [53].

**Figure 2:**
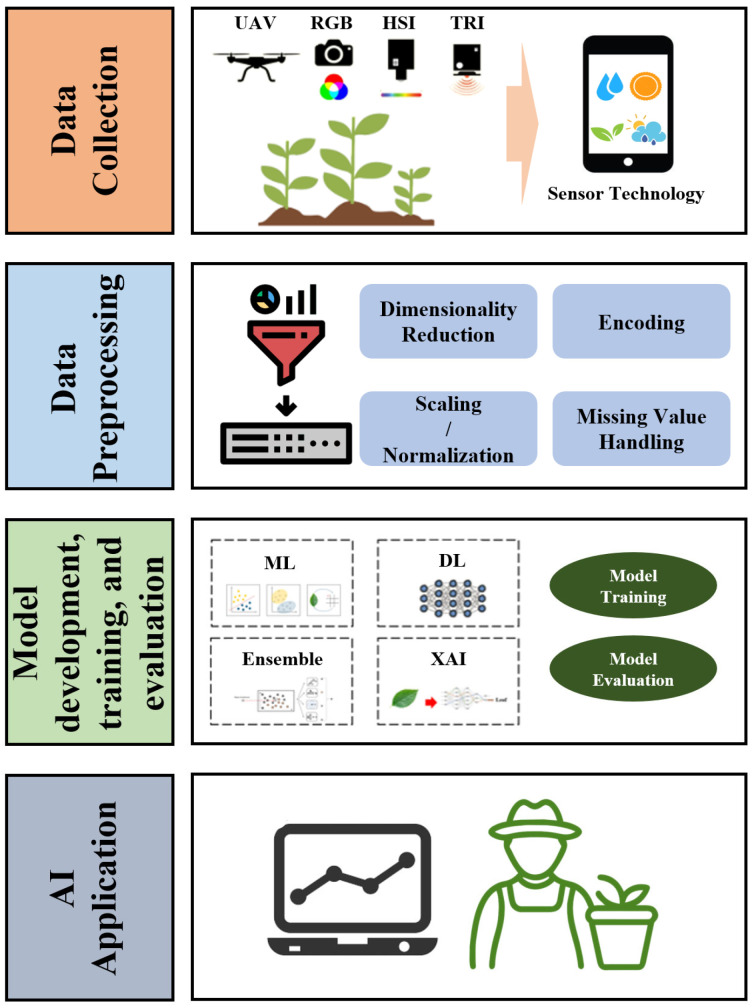
A conceptual diagram for building crop yield prediction AI model.

In the final stage, various AI-driven models are developed for CYP. They enable proactive management of yield variability and efficient resource optimization, thereby enhancing their practical applicability in real-world agricultural settings. To accomplish this, high-quality big data acquired from imaging devices, computing systems, geographical data, and climate information are required and cost-effective application strategies for the data should also be established. Figure 2 shows a conceptual diagram of the CYP model development stage introduced in this study.

### 3.1. Imaging Techniques for CYP

Imaging techniques for CYP have become a critical component in enhancing agricultural productivity and efficiency. Various imaging devices are employed for yield prediction, each offering unique characteristics and advantages. Representative devices include HSI, MSI, RGB imaging, and TRI. A total of 14 studies were selected that applied imaging techniques to CYP under open-field conditions. Table 2 summarizes the target crops, imaging techniques used, research objectives, countries, journals, and publication years from these studies.

As outlined in Table 2, the distribution of imaging techniques used for open-field CYP is as follows: HSI (about 35.7%), MSI (about 28.6%), RGB imaging (about 21.4%), and TRI (about 14.3%). The most frequently predicted crops were wheat, corn, rice, sugarcane, and soybean, with China and the United States being the countries where related research was most actively conducted.

HSI and MSI were predominantly conducted using drones or unmanned aerial vehicles (UAVs) to collect data from higher altitudes. In contrast, RGB imaging and TRI data were generally collected using fixed-mounted equipment or at low altitudes.

**Figure 3:**
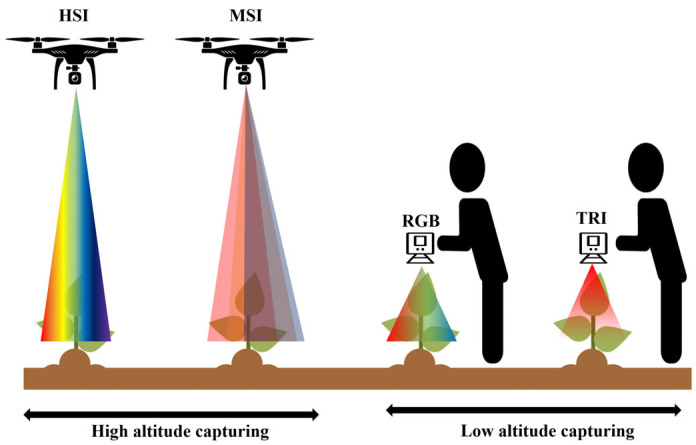
Imaging techniques used for CYP.

Notably, RGB data were rarely used independently for yield prediction; instead, they were often fused with MSI or TRI data for a more comprehensive analysis. This trend suggests that RGB imagery alone may not sufficiently capture the physiological and ecological information necessary for accurate yield prediction, and this has prompted active research efforts to integrate RGB data with other imaging modalities to enhance prediction accuracy (Figure 3).

### 3.2. ML Techniques for Open Field CYP

A total of 23 studies were selected for the development of crop yield prediction (CYP) models using ML techniques. Table 3 presents detailed information on the selected papers, including the types of ML algorithms, data sources, research objectives, and publication venues. 

In addition, the ML models summarized in the table are reported as percentages based on the frequency of their mention across the reviewed studies: Random Forest (RF) (approximately 31.6%), Support Vector Machine (SVM) (approximately 18.4%), Support Vector Regression (SVR) (approximately 18.4%), K-Nearest Neighbors (KNN) (approximately 15.8%), Artificial Neural Network (ANN) (approximately 7.9%), Decision Tree Regression (DT) (approximately 5.23%), and Gradient Boosting Regressor (GBR) (approximately 2.6%).

Among these, RF was most frequently employed for yield prediction of major staple crops such as wheat, soybean, and rice. RF has been reported to capture complex interactions among yield-related variables effectively, demonstrates robustness against overfitting, and provides stable performance across diverse growth and environmental conditions. It also offers advantages such as minimal preprocessing requirements and the ability to interpret feature importance [83]. However, most RF-based studies have been limited to specific regions (e.g., the United States, India, and China) and crop types, often relying on relatively small experimental datasets and a restricted set of environmental variables [84]. Consequently, the generalizability of these models to other climatic zones or extreme weather conditions remains insufficiently validated. Furthermore, while RF facilitates interpretation of variable importance, it may face challenges in high-dimensional, multimodal contexts (e.g., integrating remote sensing imagery with complex meteorological data), where feature selection and overfitting control become nontrivial.

SVM and SVR were the second most frequently adopted models, particularly for the quantitative estimation of rice and wheat yields in the USA, Brazil, and China. These models perform well in capturing nonlinear relationships among yield-determining variables and are often benchmarked against RF. Nevertheless, they are computationally intensive on large datasets and highly sensitive to kernel selection, which can lead to performance instability [85].

KNN and DT models were applied in studies targeting small-scale field crops in India, China, and the Netherlands. Their simple structure allows development with relatively few variables and small sample sizes, offering ease of interpretation. However, their predictive performance tends to degrade when applied to larger or noisier datasets, and results may become unstable in regions with substantially different climatic or management practices [86].

ANNs and GBRs exhibited promising potential when sufficient training data were available, but their adoption has been relatively limited. Agricultural field data are often affected by missing values, class imbalance, and measurement noise, which hinder the training stability of such models. In addition, their black-box nature constrains interpretability, which is a critical requirement for agricultural decision-making [87]. Although recent image-based crop classification studies highlight the potential of ANNs to extract patterns from unstructured data, interpretability of the factors driving predictions remains essential in the agricultural context [88].

Overall, the performance of ML-based CYP models varies considerably depending on crop type, cultivation environment, and data characteristics (e.g., sample size, number of features, temporal versus spatial focus). RF and SVM tend to perform well in settings with smaller, well-defined datasets, whereas DL or hybrid approaches are more suitable for modeling complex growth dynamics under diverse environmental stressors. Recent studies increasingly favor multi-model comparisons and ensemble learning strategies, integrating multimodal data such as climate, soil, and remote sensing to enhance predictive robustness and accuracy [89]. Nonetheless, practical challenges remain, including differences in spatial–temporal resolution across heterogeneous datasets, limited availability of extreme climate event data, and data quality issues. Future research should address these limitations by developing context-specific model selection guidelines, improving data fusion methodologies, and enhancing XAI to balance predictive performance with interpretability.

### 3.3. DL Techniques for CYP

A total of 19 papers were selected for CYP model development using DL techniques. Table 4 shows detailed information of the selected papers, including the types of DL architectures, data types used, research objectives, frequency of use, and publication sources.

The DL models summarized in the table are Artificial Neural Network (ANN) (about 17%), Convolutional Neural Network (CNN) (about 17%), Deep Neural Network (DNN) (about 17%), Long Short-Term Memory (LSTM) (about 12.5%), Recurrent Neural Network (RNN) (about 8%), Random Forest (RF) (about 8%), Linear Regression (LR) (about 4%), K-Nearest Neighbors (KNN) (about 4%), Logistic Regression (about 4%), Regression Tree (RT) (about 4%), and Multiple Linear Regression (MLR) (about 4%). These studies predominantly focus on field crops such as corn, wheat, barley, and rice. Geographically, research is concentrated in India, the United States, and China, where agriculture is strong and investment in AI is active. In India, DL-based yield forecasting is being adopted as a decision-support tool to address national priorities such as food security and climate resilience.

This distribution underscores the active exploration of diverse DL architectures for CYP. Among them, ANN, CNN, and DNN are frequently utilized due to their strong predictive performance, effectively capturing the complex and nonlinear patterns inherent in high-dimensional datasets, including satellite imagery, temporal weather sequences, and hyperspectral inputs [10,100]. CNNs and ANNs, in particular, are widely applied to major cereal crops such as wheat, maize, and soybean because of their capacity to learn spatial and visual characteristics of crop growth [6]. Likewise, LSTM and RNN models are employed to capture temporal dependencies [56], thereby elucidating the impact of environmental stressors—such as drought, precipitation, and temperature fluctuations—on yield over time [101].

Despite the growing prominence of DL, many studies continue to employ conventional ML models, such as RF and LR, as comparative baselines. Their simpler structure, interpretability, and reliable performance on relatively small and well-structured datasets justify this practice [43,102]. Indeed, when the number of variables is limited and environmental conditions are well-defined, ML methods often perform as well as or even better than DL models. By contrast, DL architectures tend to excel under conditions characterized by data complexity—such as high dimensionality, nonlinearity, or multi-source fusion—and in scenarios requiring modeling of intricate spatio-temporal dependencies. This distinction highlights an important consideration: the relative advantage of ML versus DL is highly contingent on the specific data conditions and agricultural contexts. For instance, when data availability is limited or computational resources are constrained, ML offers a cost-effective and accessible solution, whereas DL demonstrates superior performance in resource-rich environments with abundant heterogeneous data.

As DL models become increasingly sophisticated, issues of interpretability, scalability, and practical field applicability warrant greater attention. Recent developments, such as attention mechanisms, XAI frameworks, and hybrid models integrating spatial and temporal features, represent promising avenues not only for improving predictive accuracy but also for enhancing user trust and adoption [103,104]. Nevertheless, several challenges remain unresolved. DL approaches heavily rely on large-scale, high-quality datasets and substantial computational infrastructure, limiting their applicability in regions lacking such resources. Furthermore, most studies are conducted in specific countries or controlled environments, which raises concerns regarding generalizability across diverse agricultural systems and climatic conditions.

Taken together, while DL holds considerable promise for advancing CYP, particularly under complex and data-rich conditions, ML remains a highly relevant and often superior alternative in resource-constrained or well-structured environments. A nuanced understanding of these complementary strengths will be essential for aligning algorithmic development with real-world agricultural needs.

### 3.4. Ensemble Learning Techniques for CYP

Ensemble learning is a method that combines multiple learning algorithms to build a more robust and accurate model [105]. By aggregating the outputs of weak learners, ensemble learning methods can improve both predictive accuracy and model stability [106]. Common approaches include bagging, boosting, and stacking, each offering advantages in performance, robustness, and flexibility [107]. While traditional ML models often rely on a single algorithm and are prone to overfitting or data distribution issues, DL requires extensive data and computational resources [31]. In contrast, ensemble learning techniques mitigate these limitations by integrating multiple models, including both ML and DL, without depending on a single framework [108,109]. In summary, ensemble learning enhances both the performance and reliability of predictive models by leveraging the strengths of diverse algorithms [110,111].

A total of 10 papers published in the past five years were selected for open-field CYP model development using ensemble learning techniques. Table 5 presents detailed information including the types of ensemble methods, research objectives, usage frequency, and publishers.

As seen in Table 5, the predominant ensemble learning approach used for open-field CYP was stacking, incorporating both ML and DL models. Among them, ML-based ensemble learning was the most commonly applied approach. The main crops analyzed across the studies included corn, wheat, barley, rice, and soybeans.

Bagging and boosting are both widely used ensemble learning techniques; however, they have been less commonly applied than stacking in crop yield prediction studies using agricultural data. This is because bagging and boosting primarily focus on combining models based on the same algorithm, whereas stacking integrates models with different structures, thereby maximizing predictive performance [121,122].

Stacking is the most frequently adopted technique because it maximizes predictive performance by combining heterogeneous models [109]. The structure of stacking is particularly well-suited for complex tasks such as CYP, where diverse factors including climate, soil conditions, and time-series crop growth data are intricately interrelated, as it allows for the complementary reduction in both bias and variance [122].

Moreover, stacking represents an advanced approach that integrates heterogeneous learning paradigms, such as ML and DL, to enable the design of hybrid models optimized for specific data characteristics and research objectives. Traditional ML methods offer rapid training and relatively high interpretability when applied to structured numerical data; however, they are limited in capturing complex spatio-temporal interactions or processing unstructured data. In contrast, DL excels at extracting intricate nonlinear patterns from high-dimensional and unstructured inputs but entails substantial demands for large-scale training data, significant computational resources, and often suffers from a lack of interpretability [123]. Due to these contrasting properties, relying on a single paradigm is insufficient to ensure optimal performance across diverse agricultural data environments. Stacking holds considerable potential to address these complementary strengths and weaknesses; nevertheless, it also introduces challenges, including increased structural complexity, risks of overfitting, and diminished interpretability. Future research should therefore focus on developing regularization strategies and explainable ensemble frameworks to mitigate these issues.

### 3.5. XAI Techniques for CYP

XAI refers to technologies and methodologies that make the decision-making processes of AI systems understandable to humans [124]. Its primary goal is to provide transparency regarding why a specific decision was made and on what basis [35]. Key benefits of XAI include enhancing model transparency, increasing user trust, supporting decision-making, and facilitating model improvement [125].

However, limitations such as increased complexity, potential performance trade-offs, and limited explanatory power have also been noted [126]. To address these challenges, adopting simplified models or hybrid approaches that balance performance and interpretability can be effective [127].

According to the reviewed studies, applying XAI to open-field CYP enables models to identify the extent to which environmental factors such as climate and soil influence yield, thereby making the predictions more useful and actionable for agricultural management and decision-making. [38,128].

For example, Jagan Mohan et al. proposed a novel approach to understanding the complex interactions between climate and agricultural factors by utilizing XAI techniques to predict crop yields and analyze the impacts of abnormal climate [129].

Venugopal et al. conducted a comparative analysis of various XAI techniques to interpret the decision-making process of CNN models for soybean yield prediction using Sentinel-2 satellite imagery. In particular, they examined how each XAI method identifies the model’s focus on soybean fields compared to other crops or land uses [37].

Srinivaasu et al. proposed a model that classifies suitable crops based on soil and environmental conditions by applying precision agriculture and XAI techniques [104].

Kumar et al. proposed a hyperparameter optimization-based grid search algorithm and utilized XAI to enhance the transparency and interpretability of the model [130].

Martindhl et al. introduced an XAI-based smart agriculture system that provides comprehensive crop recommendations for precision farming, aiming to simultaneously improve productivity and reduce environmental impact [128].

Malashin et al. optimized the hyperparameters of a DNN model using a genetic algorithm (GA) based on climatic and agricultural variables (such as crop type, season, and region) in India, and applied LIME-based XAI techniques to analyze model interpretability and identify key influencing features such as crops [103].

**Figure 4:**
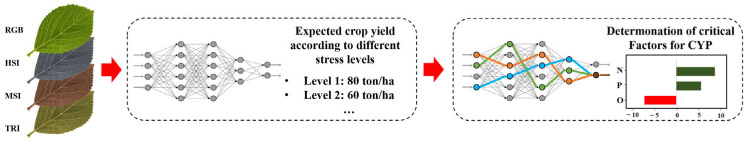
Conceptual diagram for XAI application process based on CNN model.

Based on previous studies, integrating AI and XAI into CYP plays a crucial role in agricultural adaptation to abnormal climate [131]. Accurate yield forecasts help farmers plan planting, choose crops, and manage irrigation to reduce climate risks [132]. Additionally, policymakers can utilize these predictions to develop strategic plans for food security, resource allocation, and disaster preparedness. Furthermore, the explainability provided by XAI ensures that these predictions are not only accurate but also actionable [133] (Figure 4).

Farmers and agricultural advisors can understand the rationale behind the predictions. This allows them to make informed decisions that align with local conditions and sustainability goals [134]. This approach fosters a data-driven agricultural ecosystem built on reliable and transparent AI insights.

## 4. Environmental Factors Affecting Crop Yields

**Figure 5:**
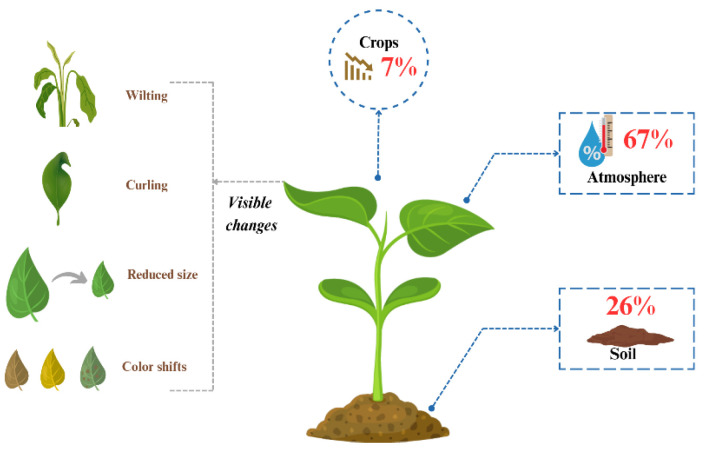
Visualization of environmental factors mentioned in CYP.

Accurate CYP requires the identification of key environmental factors that are closely associated with yield outcomes. Since these factors fundamentally determine productivity, precise measurement of environmental variables is critical to ensure optimal prediction performance [135]. In this section, we systematically categorize and summarize the major environmental factors reported in the studies reviewed in Section 3.1, Section 3.2, Section 3.3 and Section 3.4. This synthesis highlights the extent to which climate-, soil-, and crop-related variables have been employed and examines their potential influence on CYP performance. The analysis indicates that the environmental factors identified in this review serve as primary determinants of crop growth and can be broadly classified into three categories: crop-related, soil-related, and temperature-related factors. The environmental factors identified in this study were found to be key determinants of crop growth and were categorized into three main groups: crop-related, soil-related, and temperature-related factors. The most influential factors were temperature and soil conditions—specifically temperature, precipitation, soil moisture, and fertility, followed by average temperature, humidity, land area, leaf temperature, and crop length. The factors affecting each crop’s yield varied slightly, but most showed a strong influence of climatic conditions, such as temperature and precipitation. Additionally, we visualized their relative contributions to CYP performance in Figure 5.

Environmental factors serve as the foundation for crop development, with their significance and mechanisms of impact differing based on crop type and regional characteristics [136]. Variables such as temperature and soil directly affect core physiological processes and are indispensable for yield prediction [137].

For instance, temperature affects key metabolic activities including photosynthesis, respiration, and water absorption, while extreme temperatures can negatively impact crop growth [80]. Furthermore, soil, water, and nutrient supply are crucial aspects and accordingly soil fertility and moisture content are significant factors influencing crop yield [81]. However, the importance of these factors may vary depending on the local climate and the specific characteristics of the crops [138,139].

The accuracy and reliability of predictions depend on how effectively the system can handle a variety of environmental conditions [140,141]. If the key environmental factors are accurately incorporated into the model, its accuracy and reliability will improve, leading to better predictions of actual crop yield variability [142,143]. Furthermore, the higher the quality and consistency of the data are for the most significant environmental factors, the more reliable the prediction results will be. Therefore, we conclude that integrating key environmental factors as input features in predictive models is essential to improve the performance of CYP.

## 5. Other Factors of Crop Yield Decline

The most important factors affecting yield reduction are water stress and abnormal climate caused by temperature increases and irregular precipitation patterns [144,145,146]. In addition to climate-related issues, Section 4 provides a summary of other major environmental variables that can lead to decreased yields. This study further explores different factors, extending beyond abnormal climate, that can potentially influence crop yield.

The other major causes can be broadly categorized into two groups: (i) pests and soil fertility degradation and (ii) economic factors along with a lack of agricultural infrastructure [147]. A primary contributor within the former group is the presence of pests and declining soil fertility. Pests and plant pathogens directly threaten crop cultivation [148]. Although methods such as chemical and biological pest control, as well as genetically modified crops (GMOs), are employed to mitigate these threats, considerable damage can still occur [149,150]. Additionally, approaches such as chemical control can lead to soil fertility degradation due to excessive use of chemical fertilizers, which ultimately hampers plant growth by preventing crops from obtaining essential nutrients [151].

The other primary cause of declining crop yields is insufficient agricultural infrastructure, which can also lead to reduced incomes for the agricultural regions [152,153,154]. Rising costs of agricultural inputs, labor shortages, and insufficient irrigation facilities prevent farmers from securing the necessary resources for crop cultivation [155,156]. In developing countries, these challenges further reduce crop yields, with limited access to essential agricultural inputs and technical support exacerbating the reduction in yield [157,158].

In conclusion, the decline in crop yields results from the combined effects of climatic variables, biotic stress, soil fertility degradation, and structural limitations within agricultural systems. However, existing responses often address these factors in isolation or rely on short-term measures, lacking an integrated approach [159]. In vulnerable regions, the absence of context-specific strategies and insufficient coordination between policy and research further limit tangible improvements [160]. Therefore, beyond climate-resilient farming practices, targeted resource allocation, and policy-driven investment, structural reforms tailored to regional characteristics are urgently required.

## 6. AI-Based CYP: Status, Challenges, and Prospects

Currently, AI-based CYP technologies are transitioning from the research stage to partial commercialization, with some services becoming available for public use. Several agricultural technology companies, primarily based in the USA, India, and Europe, have developed and deployed AI-powered solutions that analyze satellite imagery, weather data, and soil information to estimate crop conditions and CYP [161,162]. BR Prakash et al. proposed a model that considers rainfall as a parameter along with temperature, humidity, and soil moisture content. Based on the amount of rainfall, the model determines whether to initiate irrigation. A Global System for Mobile Communications (GSM) module is used to alert farmers in the event of heavy rainfall or severe drought conditions. Additionally, a security feature is enabled, which includes the encryption component of the Wi-Fi module [163].

However, several practical challenges remain before these technologies can be widely adopted across diverse agricultural contexts. First, limited access to high-quality, large-scale data in some regions can reduce model accuracy, particularly in developing countries or rural areas lacking data infrastructure. Second, for AI-generated predictions to be trusted and interpreted by farmers and policymakers, model explainability should be improved. To overcome these challenges, governmental support including public investment in data infrastructure, AI training programs, and field-oriented user interfaces is necessary [164,165]. A key strategy to make these technologies more practically applicable could be establishing reliable public-sector prediction services that directly connect with local farming communities [166].

Enhancing the accuracy of AI-based CYP requires several strategic steps. Primarily, it is crucial to integrate multimodal data, including variables such as climate, soil, pests, and crop varieties [167]. In addition, advanced model architectures such as LSTM, Transformer networks, and hybrid ensemble learning models should be adopted to capture both temporal and spatial dynamics [168,169]. The integration of XAI techniques will also be vital for increasing user trust [129]. Beyond technological improvements, building a collaborative ecosystem among policymakers, farmers, and technology providers will be essential to promote the real-world adoption and impact of AI in agriculture.

## 7. Conclusions

CYP is recognized as a critical task in the agricultural sector, as it plays a vital role in responding to climate-induced environmental changes and enhancing the quality of agricultural products. This study comprehensively explores the applicability of various AI techniques including ML, DL, ensemble learning, and XAI for CYP. In addition, this study analyzed the imaging techniques used in CYP, the key environmental factors affecting yield, and other major causes of yield reduction.

The analysis showed that selected studies employed diverse features depending on research objectives and data availability. Despite the unified focus on AI-based CYP, the analyzed literature demonstrated considerable variation in the features used. Differences were also evident in study scale, target region, and crop type. Feature selection was primarily influenced by data accessibility and specific research goals. Importantly, including more features did not necessarily improve prediction accuracy; instead, stepwise evaluation of various feature combinations was found to be more effective in optimizing model performance.

Various algorithms were implemented across the different studies. Although it was difficult to identify the model that provides the best performance for CYP, the primary ML models observed in this review can be summarized as follows. Among ML models, RF and SVR were the most commonly applied, while ANN and CNN were widely adopted in DL. In ensemble learning, stacking techniques combining multiple ML algorithms were the most prevalent. Although existing studies on XAI are still limited, it holds significant potential for effectively addressing complex and multifactorial variables in CYP. With regard to imaging techniques, HSI and MSI were the most frequently used, primarily deployed via drones or UAVs. In contrast, RGB imaging was rarely used independently and was typically integrated with MSI or TRI.

AI-based crop yield prediction faces multiple structural challenges under abnormal climate conditions. Extreme events such as heatwaves, droughts, and irregular rainfall induce distribution shifts that weaken model generalizability, while the scarcity and imbalance of extreme-event data further exacerbate predictive uncertainty. In addition, integrating multimodal data of differing formats and resolutions—such as climate, soil, and imaging information—introduces challenges related to spatiotemporal alignment, missing values, and noise. Models are often trained on data concentrated in specific regions and crop types, making transferability to other contexts difficult. Nevertheless, the studies reviewed in this paper, all of which directly or indirectly address abnormal climate conditions, demonstrate that AI models can maintain relatively high predictive performance under such scenarios (reported performance: RMSE 0.2–0.5 t/ha, R^2^ 0.4–0.9). In other words, while the potential of AI for yield prediction under abnormal climate has been substantiated, significant region- and crop-specific limitations and uncertainties remain.

Therefore, future research should prioritize expanding training datasets to encompass a wider range of abnormal climate events, integrating multimodal inputs (e.g., weather, imaging, and crop growth data), adopting robust learning and domain adaptation techniques to handle distribution shifts, and employing XAI- and ensemble-based methods for uncertainty quantification to further enhance predictive capability.

Major environmental factors caused by abnormal climate identified atmosphere and soil conditions as the most influential variables affecting yield. The primary causes of yield reduction were broadly categorized into three groups: abnormal climate, pest, and disease outbreaks; declining soil fertility; and economic factors including limited agricultural infrastructure.

In conclusion, this study provides a comprehensive overview of the potential applications of AI, sensor technologies, and imaging methods in CYP. The findings are expected to contribute to the development of precision agriculture and smart farming systems. Furthermore, by identifying the key environmental factors and variables influencing prediction accuracy, this study can support the design of customized prediction models and inform data-driven agricultural policymaking.

## Figures and Tables

**Table 1:** Distribution of papers across different databases showcasing initial and paper numbers after applying exclusion criteria.

Database	Search Terms and Criteria	Language	No. of Paper Initially Retrieved	No. of Paper After Exclusion Criteria
Google scholar	Machine learning, Deep learning, Yield prediction, Crop, Artificial Intelligence, Abnormal climate	English	90	26
Scopus	Machine learning, Deep learning, Yield prediction, Crop, Artificial Intelligence, Abnormal climate	English	76	7
Web of Science	Machine learning, Deep learning, Yield prediction, Crop, Artificial Intelligence, Abnormal climate	English	80	22
Science direct search	Machine learning, Deep learning, Yield prediction, Crop, Artificial Intelligence, Abnormal climate	English	87	5

**Table 2:** A photographic technique used to predict crop yields.

Target	Imaging Methods Used	Objective	Ref.	Publisher	Nation	Year
Corn	HSI	Training of a CNN classification model to estimate corn kernel yield using HSI	[54]	Comput. Electron.	China	2021
Sugarcane	HSI	Yield prediction of various new genotypes in Florida sugarcane breeding using UAV-based aerial imagery and ground data collection	[55]	Agronomy	USA	2022
Rice	HSI	Yield prediction accuracy of rice grown under diverse environments using UAV-based HSI	[56]	Remote Sensing	Japen	2023
Wheat	HSI	Wheat yield prediction throughout the growing season using hyperspectral reflectance data	[57]	Comput. Electron.	China	2024
Wheat	HSI	Winter wheat yield prediction using ML based on hyperspectral data collected during flowering and grain-filling stages via low-altitude UAV	[58]	Agronomy	China	2022
Wheat, Corn	MSI	Simultaneous utilization of spatial, spectral, and temporal information from multi-spectral and multi-temporal remote sensing imagery	[59]	Int. J. Appl. Earth Obs. Geoinf.	China	2021
Rice	MSI	Exploring the potential of multimodal deep learning for rice yield prediction using multispectral UAV imagery at early growth stages combined with weather data	[60]	Remote Sensing	Japen	2023
Soybean	MSI	Monitoring soybean growth to predict yield using 14 vegetation indices, including CC, NDVI, GNDVI, and EVI2	[61]	Agronomy	USA	2024
Corn	MSI	Evaluating the effectiveness of UAV-based VIs for maize yield prediction during vegetative and reproductive stages using various ML models with limited training samples	[62]	Agronomy	USA	2023
Wheat	RGB, TIR	Development of an ML model integrating thermal and RGB indices with key plant biophysical parameters to improve yield prediction accuracy	[63]	Scientific Report	India	2023
Wheat	RGB, TIR	Exploring the potential of improving grain yield prediction by fusing source-sink level color, texture, and temperature features extracted from RGB imagery with thermal images from proximal sensing technologies	[64]	Food Energy Secur.	China	2022
Wheat	RGB, MSI	Evaluating the effectiveness of multimodal data fusion using UAV-based time-series remote sensing data and RGB and multispectral sensors for estimating wheat yield, biomass, and straw–grain ratio	[65]	Biosystems Engineering	China	2023
Corn	TIR	Improving the effectiveness of thermal imaging for assessing water stress and predicting yield in maize	[66]	J. Agron. Crop. SCI	Thailand	2022
Wheat	TIR, MSI	Fusion of UAV-based multispectral and thermal infrared data for wheat yield prediction	[67]	Agriculture	China	2022

**Table 3:** ML techniques for yield forecasting and highlights.

Target	Best Model	Quantitative Performance Metrics	Methodologies	Objective	Ref.	Publisher	Nation	Year
Wheat	SVR	R^2^: 0.77 RMSE: 0.55 t ha^−1^	SVR	Model evaluation for wheat yield prediction	[68]	ISPRS J.	France	2020
Barley Canola	RF	RMSE: 0.36 to 0.42 t ha^−1^ Lin’s concordance correlation coefficient: 0.89 to 0.92	RF	Yield forecasting for wheat, barley and canola crops	[69]	Precis	Australia	2019
Potato Corn	SVR	R^2^: 0.857	RF SVR	Potato and maize yield prediction based on weather monitoring (precipitation, temperature)	[70]	Remote Sensing	Irish	2023
Soy bean	RF	MAE: 0.42 Mg ha^−1^	SVR SVM RF	Seasonal Soybean Yield Forecast	[71]	Meteorol.	Brazil	2019
-	SVM	Accuracy: 0.88~0.90	SVM KNN RF	Soil crop yield prediction study	[72]	Food Qual.	India	2022
Maize Potatoes Rice (Paddy) and wheat	RF	R^2^: 0.96	GBR RF SVM DT Regression	Crop yield prediction research	[73]	ICCES	India	2021
Potato	SVR	RMSE: 5.97, 4.62, 6.60, 6.17 t/ha	RF SVR KNN	Potato tuber yield prediction from soil and crop characteristic data	[74].	Agronomy	Canada	2020
Rice	RF	RMSE: 0.085 R^2^: 0.93	ANN SVR KNN RF	Evaluate the most necessary features for yield prediction	[75].	Appl.	India	2019
Rice Maize Cassava Seed Cotton Yams Banana	DT	R^2^: 0.95	DT Regression SVR KNN	Yield predictions for six crops	[76]	Smart Agric.	Afirca	2022
Ceres-Wheat OilcropSun	RF	RMSE: 0.35–0.38	KNN RF ANN	Selection of a prediction algorithm and evaluation of data partitioning strategies on RF performance	[77]	Front. Plant Sci.	Netherlands	2023
Potato	RF	R^2^: 0.75–0.79	RF SVM SVR	Yield predictions for potato	[78]	Remote Sensing	USA	2021
-	SVM	Accuracy: 0.97 Error Rate: 0.05	SVM RF DT	Presenting a ML-based framework for crop yield prediction	[79]	ICIRCA	USA	2021
Wheat	RF	R^2^: 0.75	SVM RF ANN	Wheat yield forecast across Australia	[80]	Meteorol	USA	2019
Rice	SVM	RMSE: 737 kg/ha R^2^: 0.33	KNN SVM RF	Comparison of MLR and ML techniques	[81]	Ecol. Indic	China	2021
Wheat	RF	R = 0.909, nRMSE = 18%, MAE = 0.182	ANN RF	Wheat yield prediction based on temperature variation	[82]	Remote Sens.	Pakistan	2024

**Table 4:** DL techniques for yield forecasting and highlights.

Target	Best Model	Methodologies	Quantitative Performance Metrics	Objective	Ref.	Publisher	Nation	Year
Wheat	RNN	RNN LSTM GNN	RMSE:0.496 t/ha	Wheat yield prediction by integrating remote sensing and weather forecast data	[90]	ProQuest	China	2024
Tomato Potato	LSTM	RNN LSTM Cnn MLP RF	R^2^: 0.97–0.99	Tomato and potato yield prediction using historical data including climate, irrigation schedule, and soil moisture	[91]	Remote Sens.	Portugal	2021
Soybean	CNN	Decision Tree CNN LSTM	R^2^: 0.864 RMSE: 4.803	CYP for soybean across the United States using ConvLSTM and 3D CNN	[92]	Expert Systems with Applications	USA	2021
Wheat Barley	CNN	CNN	MAE: 3.19–5.65%	Wheat and Barley Yield Forecasts	[93]	Comput. Electron.	Finland	2019
Coner	ANN	ANN	R^2^: 0.48 RMSE: 3.19 MAE: 26.65	Crop Yield Forecast	[94]	Applied Artificial Intelligence	Netherlands	2020
Rice	MIR	ANN MLR SVR KNN	RMSE: 0.051 R^2^:0.99	Crop Yield Forecast	[87]	Comput. Electron.	India	2019
Wheat Rice Jowar et	ANN	ANN	Accuracy: 0.95 MSE: 0.03	Crop Yield Forecast	[95]	IEEE Pune Sect.	India	2019
Wheat	RNN	RNN LSTM	Accuracy: 0.97	Improve accuracy by applying deep learning technology to ML algorithms	[42]	Conf. Ser.	India	2021
Rice	RNN	RNN	R^2^ = 0.97 RMSE: 0.03	Predicting Crop Yield Using Nonlinear Parameters	[96]	Comput. Mater.	India	2022
Coner	DNN	DNN	RMSE: 8.21 R^2^: 0.91	Crop Yield Forecast	[43]	Springer Proc.	USA	2019
Soybean	CNN	CNN LSTM	RMSE: 329.53 kg/ha	Soybean Yield Forecast	[97]	Sensors	China	2019
Soybean	DNN	DNN	R^2^: 0.72 RMSE: 15.9%	Aperture grain yield prediction within a DNN framework	[98]	Remote Sens.	USA	2019
Coner	LSTM	LSTM	RMSE: 1.47 mg/ha	County-Level Corn Yield Forecast	[99]	Glob. Chang. Biol.	China	2019

**Table 5:** Ensemble techniques for yield forecasting and highlights.

Target	Main Category	Used Models	Quantitative Performance Metrics	Objective	Ref.	Publisher	Nation	Year
Wheat Barley Rapeseed	Stacking	Stacking Regressor (SR) Voting Mean (VM)	R^2^: 0.79–0.89 RMSE: 7.2–8.1%	Yield estimates for three major winter crops	[112]	GIScience Remote Sens.	Germany	2024
Grape	Stacking	CatBoost RF GBDT	R^2^: 0.7504 RMSE: 0.0245 m^3^/m^3^	Grape yield prediction according to seasonal drought	[113]	Integrative Agriculture	China	2022
Soybean	Stacking	KNN SVR RF	R^2^: 0.93 MAE: 117.89 RMSE: 155.59	Soybean yield prediction and feasibility verification	[89]	Sci. Agric. Sin.	China	2023
Coner	Stacking	Lnear regression LASSO regression Extreme Gradient Boosting	RMSE: 9.5%	Predicting corn yield by taking into account the weather within the season	[114]	Plant Sci.	USA	2020
Coner	Stacking	Extreme Gradient Boosting LightGBM Adaboost CatBoost	Accuracy: 99.32%	Corn yield prediction based on TBEL stacking model	[115]	Decis. Anal. J.	USA	2023
Alfalfa	Stacking	SVR KNN RF	R^2^: 0.874	Alfalfa yield prediction by combining three basic learners	[116]	Remote Sens.	USA	2020
Coner	-	CNN-DNN	RMSE: 8.5%	Corn Yield Forecast	[117]	Front. Plant Sci.	USA	2021
23 crop types	Boosting	Extra Tree AdaBoost Gradient Boosting XGBoost	Accuracy: 85.79	Yield prediction using EESCYP-I	[118]	Conf. Adv. Comput.	India	2022
Rice Maiz Wheat Sugarcane	Stacking	RF Gradient Boosting Elastic Net Ada Boost LR KNR	R^2^: 0.98 RMSE: 124.78 t/ha MAE: 7.20 t/ha	Crop yield prediction using climate datasets (precipitation, temperature, solar radiation) across tropical to temperate zones	[119]	Mater. Today Proc.	India	2025
Cotton	Boosting	RF + Extreme Gradient Boosting Extreme Gradient Boosting	RMSE: 0.22 MSE: 0.05 MAE: 1.23	ML-based cotton yield prediction using meteorological and soil data	[120]	IEEE	China	2024

## Data Availability

This is a review article. All data cited in this study are available in the referenced publications.

## Abbreviations

The following abbreviations are used in this manuscript:

CYP	Crop yield prediction
AI	Artificial Intelligence
ML	Machine Learning
DL	Deep Learning
XAI	Explainable AI
RF	Random Forest
SVM	Support Vector Machine
ANN	Artificial Neural Network
CNN	Convolutional Neural Network
HIS	Hyperspectral Imaging
MSI	Multispectral Imaging
GDP	Gross Domestic Product
RMSE	Root-Mean-Square Error
IoT	Internet of Thing
SLR	Systematic literature Review
EC	Exclusion Criteria
TRI	Thermal Imaging
KNN	K-Nearest Neighbors
SVR	Support Vector Regression
LR	Linear Regression
DT	Decision Tree Regression
DNN	Deep Neural Network
RT	Regression Tree
MLR	Multiple Linear Regression
LSTM	Long Short-Term Memory
RNN	Recurrent Neural Network
RMSE	Root Mean Square Error
MAE	Mean Absolute Error
